# How Do Landscape Heterogeneity, Community Structure, and Topographical Factors Contribute to the Plant Diversity of Urban Remnant Vegetation at Different Scales?

**DOI:** 10.3390/ijerph192114302

**Published:** 2022-11-01

**Authors:** Xingzhao Liu, Guimei Yang, Qingmin Que, Qi Wang, Zengke Zhang, Liujing Huang

**Affiliations:** 1 College of Landscape Architecture and Art, Fujian Agriculture and Forestry University, Fuzhou 350002, China; 2 Innovation Center of Engineering Technology for Monitoring and Restoration of Ecological Fragile Areas in Southeast China, Ministry of Natural Resources, Fuzhou 350013, China; 3 College of Forestry and Landscape Architecture, South China Agriculture University, Guangzhou 510642, China; 4 School of Modern Agriculture and Environment, Weifang Institute of Technology, Weifang 262500, China; 5 School of Ecological and Environmental Sciences, East China Normal University, Shanghai 200241, China

**Keywords:** landscape pattern metrics, community structure, topographic, diversity, richness, buffer radius

## Abstract

In highly fragmented urban areas, plant diversity of remnant vegetation may depend not only on community structure and topographical factors, but also on landscape heterogeneity. Different buffer radius settings can affect the relative importance of these factors to plant diversity. The aim of this study was to examine the relative importance of landscape heterogeneity, community structure, and topographical factors on plant diversity under different buffer radii in biodiversity hotspots. We established 48 plots of remnant vegetation in Guangzhou city, one of the biodiversity hotspots. A buffer radius of 500 m, 1000 m, and 2000 m was established around the center of each sample plot, and 17 landscape heterogeneity indices in each buffer were calculated by FRAGSTATS 4.2 software. Combined with the community structure and topographical factors, the impact factors of plant diversity under different buffer radii were analyzed by multiple regression analysis. We found the following: (1) The combined explanatory power of the three factors accounted for 43% of the species diversity indices and 62% of the richness index at its peak. The three impact factors rarely act independently and usually create comprehensive cumulative effects. (2) Scale does matter in urban landscape studies. At a 500 m buffer radius, community structure combined with road disturbance indices was strongly related to diversity indices in herb and shrub layers. The stand age was negatively correlated with the tree-layer richness index. As the scale increased, the diversity indices and richness index in the three layers decreased or increased under the influence of comprehensive factors. (3) The richness index in the herb layer was more responsive to impact factors than other biodiversity indices. Except for the herb layer, the interpretation of landscape heterogeneity for each plant diversity index was more stable than that for the other two factors. Road disturbance indices, combined with the other six landscape pattern metrics, can well indicate species diversity and richness. We suggest that the vegetation area of remnant patches within a radius of 500–2000 m should be appropriately increased to protect plant diversity, and the negative effects of road disturbance should also be considered.

## 1. Introduction

Urbanization is one of the main factors affecting plant diversity at the regional scale [1,2,3,4]. However, ecologists have avoided studying urban areas for most of the 20th Century [5]. Recently, there has been a growing research interest in studying plant diversity change caused by the acceleration of global urbanization [6,7]. Urbanization has rapidly spread across biodiversity hotspots [5,8], which often refer to urban vegetation that becomes a refuge for certain species [9]. Urban vegetation has been generally divided into three types: natural, seminatural, and cultivated vegetation [10]. Natural vegetation and seminatural vegetation, especially remnant secondary forests and urban weeds (dominated by spontaneously growing vascular species), are more likely to be a refuge for some species than cultivated vegetation, and the loss of plant diversity in these vegetation types is more likely to cause extinction debt [11]. Therefore, with the rapid development of global urbanization (68% of the global population will live in cities by 2050) [12], it is necessary to study the mechanism of the impact of urbanization on plant diversity in remnant vegetation, and to provide suggestions for protecting and restoring the high quality of urban vegetation.

There are two inconsistent views on the impact of urban landscape heterogeneity on plant diversity. On the one hand, landscape heterogeneity leads to the gradual loss of large areas of biological habitats, which directly reduces species diversity [13,14,15,16]. On the other hand, diverse habitats created by landscape heterogeneity are conducive to the settlement and survival of organisms from various sources, which support higher species diversity [17,18,19,20]. Landscape heterogeneity, with respect to area, shape, configuration, and quality of the effect, such as edge effect, cluster effect, and fragmentation effect, has been proven to influence the alpha (α), beta (β), and gamma (γ) of plant species diversity [21,22,23,24,25]. Hence, landscape heterogeneity around the habitat is one of the direct driving forces for the change in plant diversity caused by urbanization. However, how local biodiversity responds remains unclear [26,27]. Moreover, community structure, such as the height, coverage, and age of stands, has been demonstrated to affect plant diversity [28,29]. For vegetation with a community structure of three layers or more, the diversity distribution patterns of different levels are different. Topographical factors such as elevation, slope, and aspect have also been shown to affect plant diversity in specific habitats [30,31,32]. Therefore, more specifically, it is necessary to reveal the relative importance of landscape heterogeneity, community structure, and topographic factors in influencing plant diversity to protect plant diversity.

Evidently, the differences in landscape metrics and study scale can affect the changes and responses of biodiversity, but there is no clear conclusion. Most scholars consider that plant diversity change is caused by various urbanization factors at different scales [6,23,25]. On a large scale (urban, regional, and global), the urbanization process and socioeconomic factors are the dominant factors affecting urban plant diversity. On a small scale, landscape patterns and habitat conditions are the dominant factors affecting urban plant diversity [33,34,35]. Landscape heterogeneity has long been considered a key determinant of biodiversity. Previous studies have not considered the scale-dependent landscape heterogeneity of local habitats [23,27,36], but the research focus has been on the time lag in biodiversity response to landscape changes and the mechanisms causing extinction debt [37,38]. In the past, studies have addressed the biodiversity in natural forest landscapes [39], agricultural landscapes, and other types of landscapes [40]; however, studies on the biodiversity in urban landscapes are lacking in the literature. Therefore, the mechanism of the scale effect and the relative importance of landscape heterogeneity and habitat conditions on plant diversity needs to be investigated. Different landscape structure parameters, such as landscape heterogeneity, landscape connectivity, landscape complexity, and landscape matrix, may have different degrees of influence on regional biodiversity and have certain scale effects [25,41,42].

How are species richness and species diversity differentially driven by the spatial scale in landscape ecology? There is still a lack of unified conclusions about the response of different layers in stands to landscape heterogeneity. For the rich and sensitive hotspots of biodiversity on Earth, it is necessary to reveal the impact of specific landscape types on biodiversity and its mechanisms to provide a reference for the protection and utilization of sustainable biodiversity. Therefore, it is necessary to combine the method of multiscale analysis to explore the distribution patterns of biodiversity on local and even regional scales from a multidimensional perspective, and to explore the commonness and regularity of biodiversity distribution on the global scale. At the same time, research should elucidate key factors, explore the mechanisms, and provide a scientific basis for biodiversity protection and management against the background of rapid and high urbanization.

China’s urbanization has attracted worldwide attention. Approximately 13% of China’s urban land is located in these biodiversity hotspots, especially in Guangdong Province, which accounts for two-thirds of the urban land in this hotspot [43]. As the capital of Guangdong Province, Guangzhou is one of the most densely populated and highly urbanized cities, with a resident population of 14.5 million and an urbanization rate of 86.14% [44]. In addition, the city has abundant plant resources in its built-up area; there are 572 species belonging to 123 families and 386 genera of vascular plants, including 228 species belonging to 61 families and 171 genera of herbaceous plants [45]. Most of these plants are distributed in the remnant vegetation in the urban area. However, there is only 290 km^2^ of natural vegetation in Guangzhou city, which is located in reserves, fengshui forests, and scenic forests, accounting for 9.4% of the total forest area (mainly plantation area) and 3.9% of Guangzhou city [46]; grassland and artificial forests occupy most of the urban vegetation. In this study, we considered remnant vegetation with less human disturbance scattered in different urban landscape patterns as research objects, and explored how landscape heterogeneity, community structure, and topographic factors affect plant diversity under different buffer radii in urbanization landscapes.

## 2. Data and Methods

### 2.1. Study Area

Guangzhou is at the heart of the most-populous built-up metropolitan area in mainland China, and is ranked as an alpha global city. It has 11 districts with an administrative area of 7434.4 km^2^. Its permanent population is 14.5 million, and its permanent population density is 1950 person/km^2^ [44], which is highly concentrated in central built-up areas. The study area is focused on the central built-up part of Guangzhou city. This core area is composed of two old districts (Yuexiu and Liwan), three medium-age developing districts (Baiyun, Tianhe, and Huangpu), and one young district (Luogang). The city’s green rate of built-up area is 37.5%, and the forest stock volume is 17 million m^3^ [47]. Guangzhou is located in the north subtropical-humid climatic zone, which is influenced by the regional dominant monsoon weather. The mean annual precipitation attained is 2035.2 mm, and the mean air temperature is 22.6 °C [44]. The mild climate enables vegetation to continuously grow all year round. The natural vegetation in this area before urbanization was subtropical evergreen broad-leaved forests, which have been destroyed by several millennia of agricultural activities and recent urbanization. The different original natural setup, development history, urban fabric, habitat conditions, and management systems have resulted in an uneven spatial pattern of urban vegetation among land uses and administrative districts [48]. In southern China, the climax vegetation is subtropical evergreen broadleaved forests.

### 2.2. Sampling and Vegetation Survey

**Figure 1:**
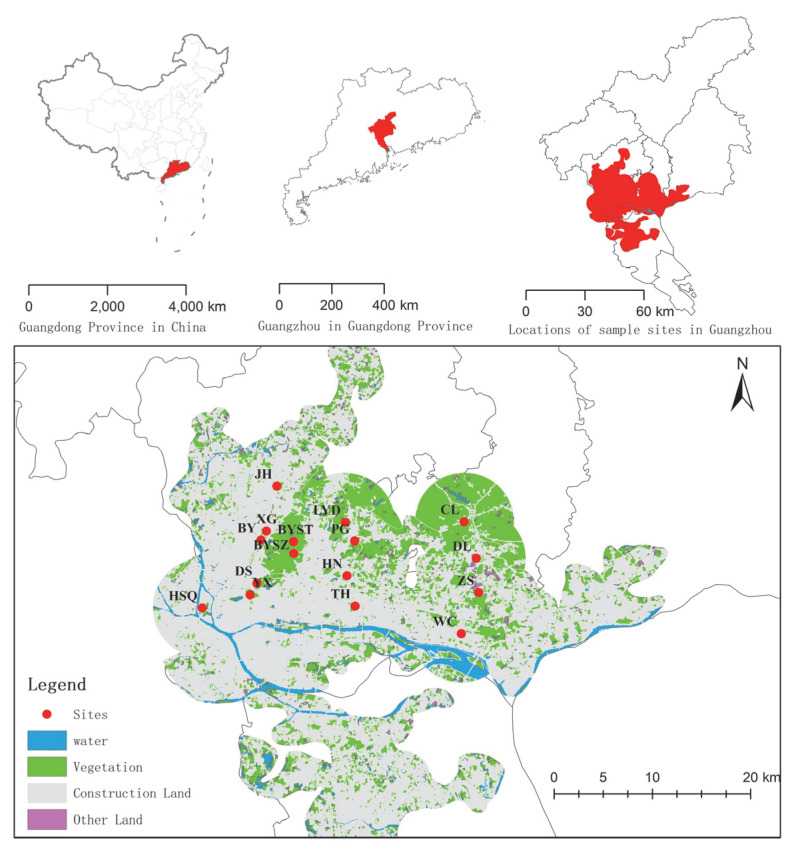
Map of 16 sample sites in different landscape patterns in Guangzhou, southern China.

To highlight the impact of urbanization (nonhuman subjective selection) on vegetation, remnant vegetation dominated by spontaneously growing vascular species was selected, including 3 types: remnant secondary coniferous and broad-leaved mixed forests, broad-leaved forests, and urban weeds. The sample sites were selected based on satellite images, land use status maps, and field surveys. A total of 16 sample sites under different landscape patterns in Guangzhou (Figure 1 and Table 1), including 4 urban weeds, 3 secondary coniferous and broad-leaved mixed forests, and 9 secondary broad-leaved forests, were selected.

Plant species in three sample plots (each 20 × 20 m^2^) at each study site were surveyed from March 2014 to March 2015. According to the technical guidelines for biodiversity monitoring [49], the forest observation plot should be larger than 1 ha, so we set three plots of 400 m^2^ (each 20 m × 20 m) in the sample sites for a total of 48 sample plots. The total survey plot was 1.92 ha, including 12 in urban weeds, 9 in secondary coniferous and broad-leaved mixed forests, and 27 in secondary broad-leaved mixed forests. Woody species and trees with a diameter breast height (DBH at 1.3 m) larger than 2 cm in each plot were counted and identified. Shrubs and herbs in the same plot were counted and identified in four randomly distributed 5 × 5 m^2^ subplots and four 1 × 1 m^2^ subplots, respectively. The species, numbers, height, density, canopy, and coverage of tree, shrub, and herb layers were individually counted and recorded. We used GPS for geographic coordinate positioning. The stand age, elevation, longitude and latitude, slope, and aspect were also recorded.

### 2.3. Landscape Pattern Metrics Combined with Community Structure and Topographic Factors

First, the landscape classification map of the research area was drawn based on the current land use status map (2011, 1:10,000), which was divided into six types: vegetation, residential area, road, construction land, river, and other lands (Figure 1). Taking each sampling point as the center of the circle, the landscape pattern metric within different buffer radii was calculated. The buffer radius was set to 500, 1000, and 2000 m. We used the vegetation coverage in the buffer zone, the road disturbance indices, the distance between the center of the plot and the edge of the forest, and some landscape pattern metrics to represent the landscape heterogeneity. The above indices of different radii in the buffer area were calculated by Fragstats 4.2. Through the collinearity analysis of the indices, a total of 17 indices were selected to characterize the landscape heterogeneity of different sampling plots, including matrix indices (1), road disturbance indices (2), distance from the edge of the forest (2), clustering indices (4), area-edge indices (1), edge indices (2), shape indices (2), and landscape diversity indices (3) (Table 2). Second, tree layer coverage, herb layer height, herb layer coverage, and stand age were selected to characterize the community structure. Third, elevation, slope, and aspect were selected to characterize the topographic factors.

### 2.4. Data Analysis

#### 2.4.1. Data Analysis of Sampling and Vegetation Survey

Importance value: Importance values are important indicators for measuring the position and function of a population in a community and are also the basis for calculating species diversity. In this study, the calculation method of Qian et al. was used to measure the importance values of each plant population in the community. The formula is as follows:

*IV_i_* = (*rf_i_* + *rd_i_* +*rdo_i_*)/3

where *IV_i_* is the importance value of species *i*; *rf_i_* is the relative frequency of species *i*; *rd_i_* is the relative density of species *i*; and *rdo_i_* is the relative dominance of species *i*.

Species diversity: Four indicators, the species richness index (*S*), Shannon diversity index (*H*’), Simpson’s index (*D*), and Pielou uniformity index (*J*), were used to measure the species diversity of arbor, shrub, and herbaceous in urban green space plant communities. In terms of plant diversity indices, four common alpha biodiversity indices were selected in this study (Table 3). The biodiversity indices were analyzed using R Language 2.11.0.

#### 2.4.2. Data Analysis of Landscape Pattern Metrics Combined with Community Structure and Topographic Factors

Multiple regression model analysis (stepwise regression, *p* < 0.05) was used to explore the relationships between landscape heterogeneity, community structure features, topographic factors, and plant diversity indices under 500, 1000, and 2000 m buffer radii. The explanatory power (adjusted *R*^2^) indicates the contribution of the impact of each factor to the plant diversity indices. Data were processed and analyzed in Microsoft 365 Excel and IBM SPSS Statistics 25.

## 3. Results

### 3.1. Species Composition in Different Remnant Vegetation in Guangzhou City

A total of 234 species (179 genera and 82 families) were recorded in the remnant vegetation at the 16 study sites. A total of 1277 trees (DBH ≥ 2 cm) representing 67 species (50 genera and 31 families), 141 shrub-layer species (103 genera and 53 families), and 128 herb-layer species (111 genera and 54 families) were recorded. A total of 38 species (37 genera and 19 families) were recorded in urban weeds, 110 species (98 genera and 53 families) were recorded in secondary coniferous and broad-leaved mixed forest, and 167 species (119 genera and 67 families) were recorded in secondary broad-leaved forest.

The dominant species (importance value >5%) in the three vegetation types are shown in Table 4. The dominant species in urban weeds belonged to *Gramineae* and *Asteraceae*, which were mainly composed of exotic species and generalists. *Bidens pilosa*, a common annual agricultural weed, was widely distributed in tropical and subtropical regions and accounted for more than 30% of the importance value among most urban weeds. It has a strong allelopathic effect and often forms a large area of monodominant population, resulting in a decrease in local biodiversity and a serious threat to the survival of local plants [50,51,52]. *Neyraudia reynaudian*, another dominant species, is a native grass species and is widely distributed in southern China, with developed roots, strong growth, and good resistance to stress [53]. The dominant herb-layer species in forests were native herb species, including shade or half-shade plants, and ferns that survive in forest or humid environments, such as *Eriachne pallescens, Pteris semipinnata*, and *Lophatherum gracile.* The dominant shrub-layer species were similar in the two different forest types, which were dominated by *Psychotria rubra.* The dominant tree-layer species in secondary coniferous and broad-leaved mixed forest was *Pinus massoniana,* while in secondary broad-leaved forest, it was *Schima superba*.

### 3.2. Plant Diversity in Different Layers of Remnant Vegetation in Guangzhou City

**Figure 2:**
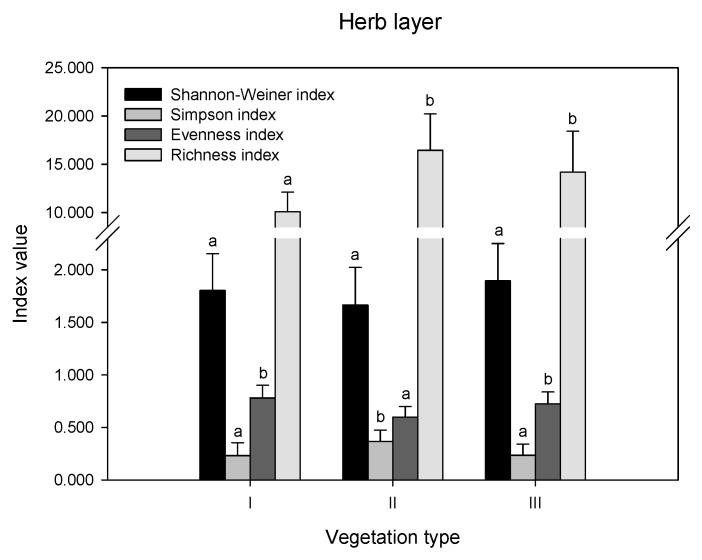

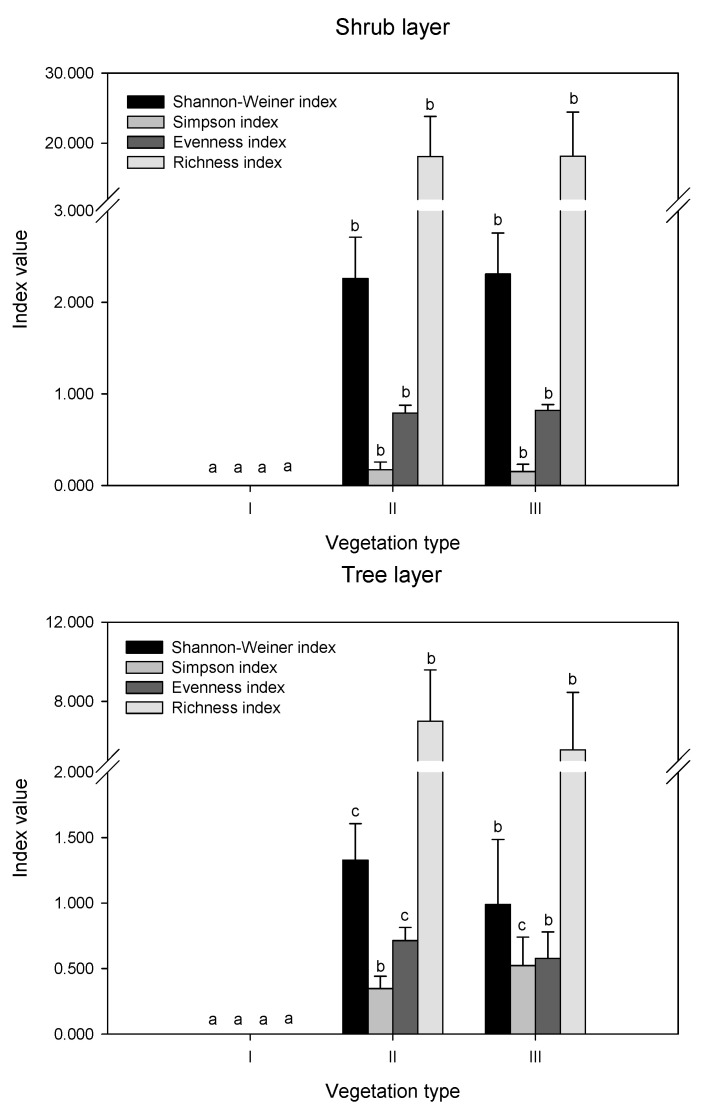
Biodiversity indices in different layers of remnant vegetation. I indicates urban weeds; II indicates secondary coniferous and broad-leaved mixed forest; III indicates secondary broad-leaved forest. Different letters indicate significant differences at the confidence level of *p* < 0.05 among the vegetation types (one-way ANOVA, *p* < 0.05).

The differences in diversity indices and richness index were compared in remnant vegetation (Figure 2). In the herb layer, there were no significant differences in the Shannon index values among the three vegetation types (*p* > 0.05). The Simpson index was highest in II, while the Evenness index showed the opposite trend, which was highest in I and III. The order of the richness index values was II > III > I. In the shrub layer, there were no differences in the values of all indices (*p* > 0.05). In the tree layer, the order of the Shannon index, evenness index, and richness index values was II > III, while the Simpson index showed the opposite trend.

### 3.3. Relationships between Landscape Heterogeneity, Community Structure, Topographic Factors, and Plant Diversity Indices

Although diversity indices and richness indices in tree, shrub, and herb layers were affected by various factors and showed different trends under three buffer radii, they still had the following common characteristics: (1) At the 500 m scale, the diversity indices in the herb layer and shrub layer were primarily affected by community structure (herb layer coverage and tree layer coverage, respectively), followed by road disturbance indices, while the diversity indices in the tree layer had no relationship with any factors. (2) As the scale expanded, diversity indices in the herb layer declined under the influence of comprehensive factors. In contrast, the richness index in the herb layer increased with increasing buffer radius, which was affected by comprehensive factors. Shrub layer diversity was affected by different factors under different buffer radii. The richness of the shrub layer decreased with increasing scale and the influence of comprehensive factors. The diversity of the tree layer was most significantly affected by comprehensive factors on the 1000 m radius scale. The tree layer richness index was negatively correlated with stand age and was affected by comprehensive factors as the buffer radius increased.

Based on the multiple regressions (Table 5), we found that the relationships between plant diversity indices and richness index and impact factors in the tree, shrub, and herb layers were significantly different with the change in scale. The plant diversity indices in the herb layer were most closely related to impact factors at the 500 m scale, and their association with factors decreased as the scale increased. The herb-layer diversity indices were mainly impacted by Ch and were also affected by road disturbance (AD, PD, and RD) at the 500 m and 1000 m buffer radii. In contrast, the richness index in the herb layer was most closely related to impact factors at the 2000 m scale, and its association with factors decreased as the scale declined. The herb-layer richness index was significantly impacted by SHAPE and Ct at the 500 m scale, and as the scale increased, the association with SLOPE, ELEVATION, LPI, and SHDI also increased.

The shrub-layer diversity indices were mainly impacted by Ct. In addition, they were also affected by road disturbance (RD and FD) at the 500 m scale; by topographic factors (SLOPE), matrix (Cv), and landscape diversity indices (SHDI) at the 1000 m scale; and by topographic factors (SLOPE), clustering indices (LPI), and area-edge indices (LSI) at the 2000 m scale. The shrub-layer richness index was significantly impacted by road disturbance (RD, AD, and FD).

The diversity index in the tree layer was most closely related to Ct, PD, and AD at the 1000 m scale, while the tree-layer richness index was significantly negatively related to AGE under the three buffer radii, and was most closely related to -AGE*, AD*, and Cv at the 2000 m scale.

### 3.4. The Explanatory Power of the Regression Model

**Figure 3:**
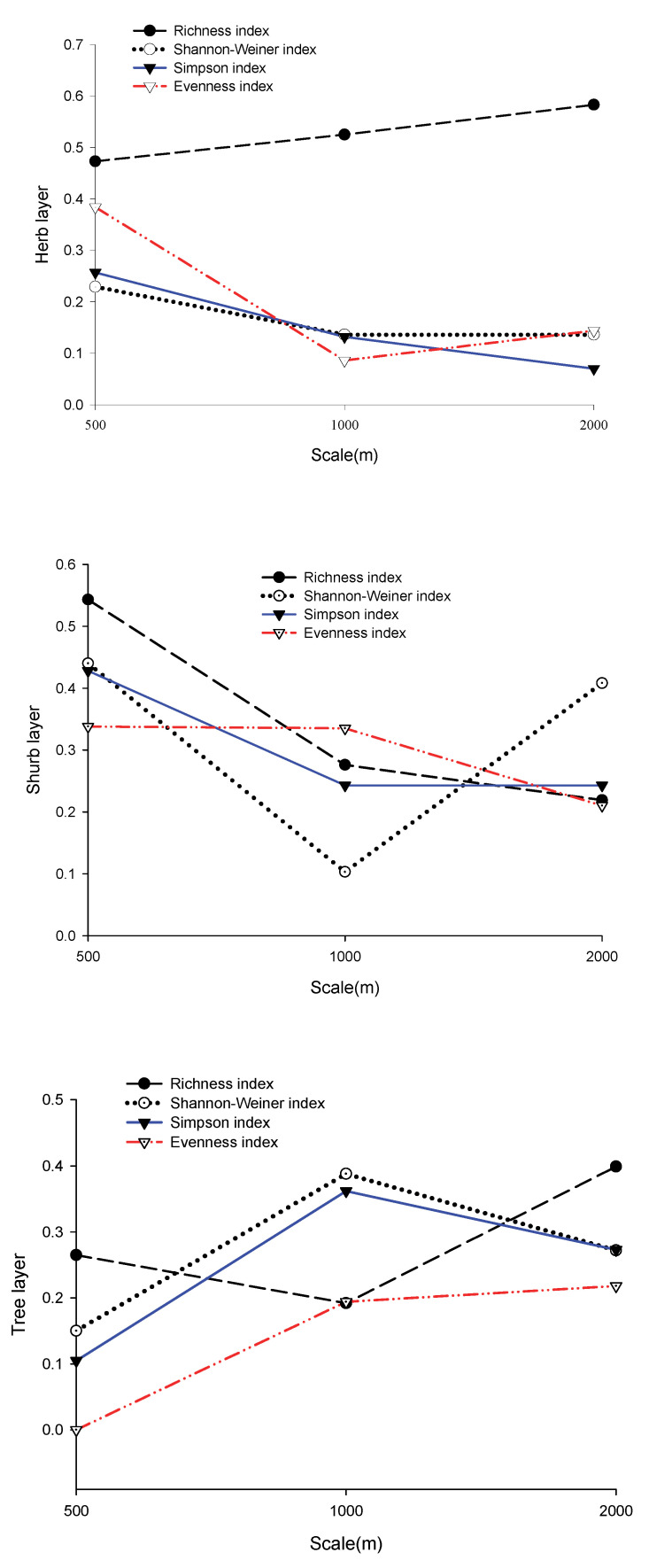
The explanatory power of landscape heterogeneity, community structure, and topographic factors on biodiversity and richness indices.

Based on the explanatory power (adjusted R^2^) of various regression models (Figure 3), landscape heterogeneity, community structure, and topographic factors had the strongest explanatory power for the richness index, but not at the same scale. The explanatory power of the three layers was ranked as follows: herb layer (0.62) at the 2000 m scale > shrub layer (0.55) at the 500 m scale > tree layer (0.40) at the 2000 m scale. The explanatory power of factors on other diversity indices was relatively weak (<0.43). The combined explanatory power of the three factors accounted for 43% of the species diversity indices and 63% of the richness index at its peak.

In the herb layer, the explanatory power of factors on the richness and diversity indices showed an opposite trend with increasing scale. The impact factor had the strongest explanatory power on the richness index for the three buffer radii (0.47–0.62), and its explanatory power increased with increasing scale. The explanatory power of factors on diversity indices (the Evenness index, the Shannon diversity index, and the Simpson index) was relatively low (0.08–0.4), and the highest explanatory power appeared on the 500 m scale. Except for the Evenness index, the explanatory power of factors on the Shannon index and Simpson index decreased with increasing scale. In the shrub layer, the regression equations of each index showed that diversity indices and richness index can both be well fitted at the 500 m scale. Except for the Shannon index, the explanatory power of factors on other indices decreased with increasing scale. In the tree layer, the explanatory power of factors on all diversity indices was shown to be the weakest at the 500 m scale. The comprehensive factor had the strongest interpretation for the Shannon index (0.39) and Simpson index (0.36) at the 1000 m scale.

### 3.5. The Relative Importance of the Effects of Landscape Heterogeneity, Community Structure, and Topographic Factors on Diversity Indices and Richness Index

**Figure 4:**
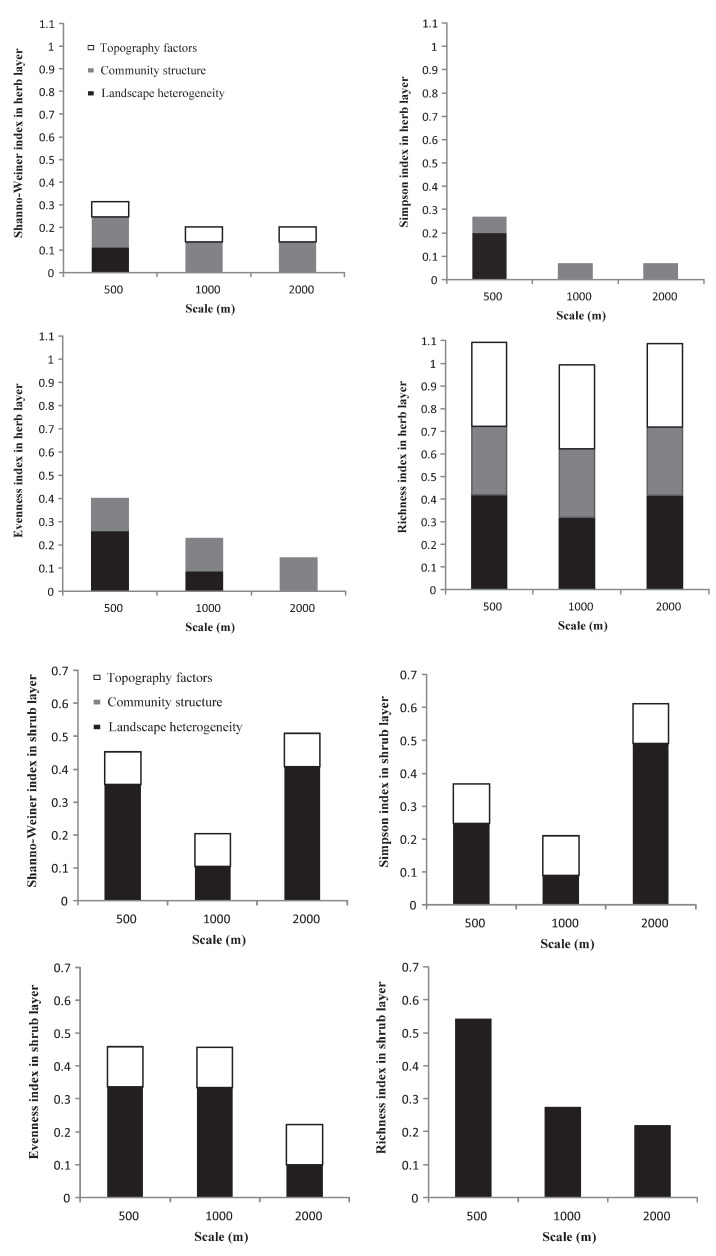

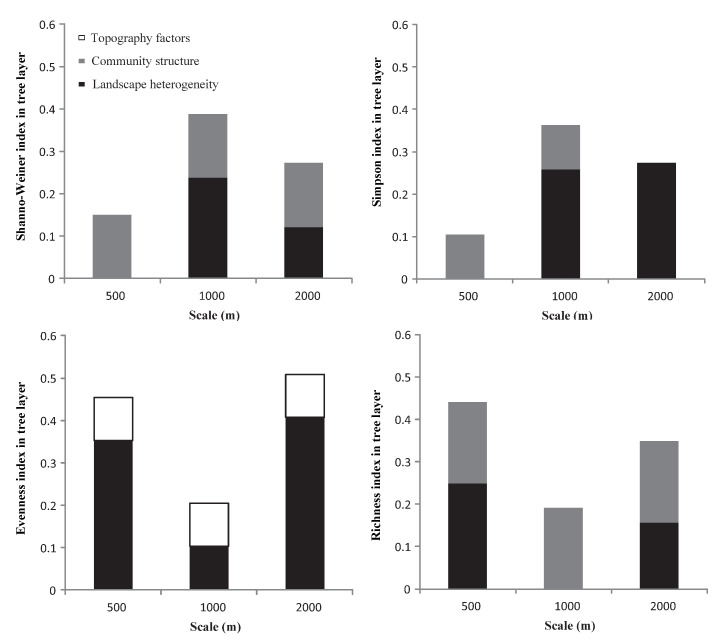
The variance decomposition of landscape heterogeneity, community structure, and topographic factors in the multiple linear regression models.

Based on the contribution of each index to the overall explanatory power of the regression model (Figure 4), and except for the herb layer, the interpretation of landscape heterogeneity for each plant diversity index was more stable than that for the other two factors. In general, the relationships between the three impact factors and the diversity indices and richness index under different buffer radii showed strong scale volatility, but lacked consistency.

In the herb layer, the explanatory power of community structure for each plant diversity index was more stable than that of the other two factors. Community structure combined with landscape heterogeneity had a relatively strong explanatory power for plant diversity indices at a 500 m buffer radius compared to the others. For the richness index, the superposition of the three factors led to a very high overall explanatory power of the model.

In the shrub layer, landscape heterogeneity had a relatively higher explanatory power for all diversity indices and richness indices across the three buffer radii than topographic factors. For the Shannon and Simpson indices, landscape heterogeneity had the highest explanatory power at the 2000 m scale. For the Evenness index, the landscape heterogeneity showed the same explanatory power at the 500 m and 1000 m buffer radii. For the richness index, landscape heterogeneity was the only explanatory power that weakened as the scale increased.

In the tree layer, except for the Evenness index, landscape heterogeneity and community structure had explanatory power for the other diversity indices and richness index across the three buffer radii. For the Shannon and Simpson indices, landscape heterogeneity combined with community structure had a high explanatory power at 1000 m. For the Evenness index, landscape heterogeneity combined with topographic factors had the highest explanatory power at the 2000 m scale. For the richness index, landscape heterogeneity combined with community structure had the highest explanatory power at the 500 m scale.

## 4. Discussion

### 4.1. The Response of the Diversity Index to the Impact Factors

From the different indices that characterize plant diversity, the diversity indices commonly used in the past, such as the Shannon diversity index (H’), Evenness index (E’), and dominance index (D’), were not well interpreted; however, the richness index in the herb layer was more responsive to impact factors than other biodiversity indices. This strong scale dependence is probably because the traditional diversity indices were also based on statistics, so the correlation is probably only statistical rather than ecological. Therefore, the richness index is more appropriate for analyzing the impact of landscape pattern changes on plant diversity. On the other hand, the richness index is additive and has the same measurement unit, so its ecological significance will be easier to understand and explain.

Therefore, the richness index can be used to effectively compare the differences in plant diversity in human activity intensity and landscape background in different spaces and temporal buffer radii [54].

### 4.2. The Relative Importance of Impact Factors on Species Biodiversity Varying with Buffer Radius

The analysis of biodiversity, without considering scale effects, may lead to erroneous conclusions [55]. Studies have shown that on a small spatial scale, changes in the environmental conditions of a community or ecosystem lead to changes in plant diversity and species composition [56,57]. However, on a large spatial scale, the effects of landscape pattern characteristics on species have not yet reached a consistent conclusion [58,59,60,61,62]. In general, our results found that the relationships between the three impact factors and the diversity indices and richness index under different buffer radii showed strong scale volatility, but lacked consistency.

According to the results of this study, the combined explanatory power of landscape heterogeneity, community structure, and topographic factors accounted for 43% of the species diversity indices and 62% of the richness index at its peak. Unexplained parts may be caused by factors that are not covered, such as spatial variation or the effects of random processes [63]. Although the factors that may affect the species diversity of urban remnant vegetation are complex and scale-dependent, and the tree, shrub, and herb layers may have different response mechanisms to them, relatively uniform research results may be obtained. Factors related to landscape heterogeneity, community structure, and topographic factors may not act alone, but they may create synergistic effects.

Our results found that as the scale increased, the richness index in the herb layer was affected by comprehensive factors that increased with increasing buffer radius. This is similar to many research results, indicating that the correlation between plant species richness and landscape pattern is enhanced with the expansion of spatial scale [42]. Species richness in the shrub layer and herb layer was under the influence of road disturbance and stand age, respectively. This may be because the species richness distribution of different functional groups is not affected by landscape structure factors, such as isolation degree, and only a few are affected by patch area, patch distance from road, and patch type. It may be that species at small buffer radii can spread to the target habitat in close proximity, and diffusion is limited to a small scale range, so isolation and other factors are not the maintenance mechanism of species richness [48,64].

### 4.3. The Impact of Landscape Heterogeneity on Plant Diversity

Some of the studies found that the relationships between landscape heterogeneity and plant species diversity are weak, and show strong scale volatility and lack consistency [65,66]. However, our results showed that the interpretation of landscape heterogeneity for each plant diversity index in shrub and tree layers was more stable than for the other two factors, which is consistent with the conclusion that buffer radii of 1000 m and 2000 m are the appropriate buffer radii to study the influence of various landscape patterns on forest, nonforest, and universal species diversity [67]. The reasons for this might be that (1) the 500 m scale is not suitable for analyzing the influence of landscape heterogeneity on the diversity in the tree layer of 20 m × 20 m patches; and (2) plants in the tree layer are mainly woody plants, whose response to landscape heterogeneity in the small-scale range is relatively lagging.

At a 500 m buffer radius, habitat conditions such as community structure and topographic factors may be the key factors determining species distribution [68,69]. However, our results showed that community structure combined with landscape heterogeneity had a relatively strong explanatory power for plant diversity indices in the herb layer at a 500 m buffer radius compared to the others. The reasons may be as follows: (1) *α* biodiversity is mostly affected by the matrix landscape characteristics around the habitat patches at the 500 m scale, which is mainly caused by adjacent human activities [67]. (2) The effect of the landscape complexity index on the diversity in the herb layer on a small scale (250–750 m) is higher than that on medium and large scales [70].

In general, the richness index and diversity index were influenced by different degrees of landscape heterogeneity (-SHAPE, -LPI, -ED, SHDI, -PD, Cv, and -LSI). Studies on tropical forests have shown that diversity indices of trees, shrubs, and lianas are mainly affected by patch shape (SHAPE) and patch diversity (SHDI) [71]. Our study found that SHAPE, ED, and LPI, which characterize the shape and edge of the landscape, had a negative impact on the richness index in the herb layer. Studies have reported that irregular landscape patches tend to maintain high plant diversity [72]. It is generally believed that narrow or irregular patches have large marginal lengths and marginal densities (ED), which lead to high landscape heterogeneity and relatively high plant diversity [42,73]. The reason may be that at different buffer radii, a higher marginal density (ED) of habitat patches helps maintain high species richness [21,74]. However, experimental studies have shown that the shape complexity index of landscape patches is inconsistent with plant diversity, or indicates either higher plant diversity, or lower plant diversity [71,73]. At the same time, studies in Western Europe have shown that plant diversity, especially woody plant species diversity, is inconsistent with different landscape shape indices [21], which may be related to landscape types and sampling scales.

With regard to landscape diversity, our study found that the SHDI was positively correlated with the herb-layer richness index. The consistent conclusion was that the species richness index is high in heterogeneous landscapes. Species richness has been reported to positively correlate with the diversity of landscape type patches [21], whose positive correlation is derived from abundant landscape landform types [75] or from landscape quality effects and adjacency effects that provide potential habitats for more species [39]. Studies in Western Europe have indicated that plant diversity, particularly the species diversity of woody plants, is significantly positively correlated with landscape diversity [21]. Moreover, studies in Austria [76], Belgium [77], and Spain [73] showed that the species richness of vascular plants, mosses, and birds positively correlated with landscape diversity. A study of vascular plant diversity in grasslands found that as the diversity of habitats around the plot increased, species diversity increased; conversely, as the habitat diversity decreased, the α diversity index decreased [78].

In addition to being related to landscape diversity, plant diversity indices are also associated with the aggregation degree between landscape patches. Regarding clustering indices (patches aggregation degree index), our study showed that patch density (PD) and largest patch index (LSI) had a negative effect on herb layer richness.

The results indicated that Cv was positively correlated with the diversity index in the tree layer. On the basis of the theory of island biogeography, in a certain range, the area of landscape patches is positively correlated with species richness and the diversity index, but this relationship weakens after exceeding a certain area [21,72]. The selective extinction hypothesis suggests that patch area is an important factor restricting species distribution; for example, the species with the smallest requirement area are more likely to become extinct in smaller patches [79]. Plant diversity has been shown to increase with the increase in habitat patch area and habitat similar to the surrounding landscape type, which would improve the habitat [80]. This is because the larger patch area contains more micro habitat types, and the amount and type can provide more species habitat. However, for different species, the relationship between landscape patch area and species richness is also different [81]. Studies on semiarid steppes have shown that plant richness and diversity indices were significantly positively correlated with patch area and habitat center area [67]. Many studies have found that the urban ecological system has a high diversity of vascular plants, especially in the green belt, of both native and exotic species, which means that the larger the patch area of greening is, the higher the diversity of vascular plants [82]. As global landscape fragmentation has become increasingly serious, small patches have become a common feature of the ecosystems, and at the same time, they are the main habitat in urban ecosystems. It is necessary to define the minimum patch area that allows native plants to survive. In many ecosystems, the minimum patch area controls the entire ecosystem. Therefore, the assessment of the size of patches in the maintenance ecosystem plays an important role in guiding researchers to determine the minimum patch area and number [83]. This is similar to many research results, indicating that species richness is significantly affected by patch area [70].

Our study showed that the road disturbance factors (- AD and - RD) and the farthest distance from the sample point to the forest edge (- FD) had different degrees of negative impact on the richness index in all three layers at different buffer radii. Roads, railways, and other artificial barrier edges often reduce biodiversity [84]. As patch area increases, the richness index of edge species and internal species increases, with the latter significantly increasing [72].

Finally, our results showed that road disturbance indices (AD and RD), farthest distance from the sample point to the forest edge (FD), area-edge indices (LPI), edge indices (ED), shape indices (SHAPE), and landscape diversity indices (SHDI and SIDI), i.e., eight indices in total, can well indicate species diversity and richness.

## 5. Conclusions

(1) The response mechanisms of the plant richness index and diversity indices in different layers under different buffer radii to impact factors were different. Compared to the biodiversity indices commonly used in the past, such as the Shannon diversity index (H’), evenness index (E), and dominance index (D’), the richness index in the herb layer was more direct and sensitive than the richness index in the tree and shrub layers and the diversity indices in the three layers to the impact factors.
(2) The combined explanatory power of landscape heterogeneity, community structure, and topographic factors accounted for 43% of the species diversity indices, and 62% of the richness index at its peak.
(3) The three impact factors that affect the species diversity indices and richness index of urban remnant vegetation rarely act alone, and often cause comprehensive cumulative effects and scale dependence.
(4) Scale does matter in urbanization landscape studies. At a 500 m buffer radius, community structure combined with road disturbance indices was strongly related to diversity indices in herb and shrub layers. The stand age was negatively correlated with the tree layer richness index. As the scale increased, the diversity indices and richness index in the three layers decreased or increased under the influence of comprehensive factors.
(5) Except for the herb layer, the interpretation of landscape heterogeneity for each plant diversity index was more stable than that for the other two factors. Road disturbance indices (AD and RD), farthest distance from the sample point to the forest edge (FD), area-edge indices (LPI), edge indices (ED), shape indices (SHAPE), and landscape diversity indices (SHDI and SIDI), a total of 8 indices, can well indicate species diversity and richness.

Under the background of rapid urbanization and increasingly fragmented urban vegetation, the buffer area of remnant patches, such as grassland and woodland, should be reasonably allocated. Furthermore, we suggest that the vegetation area of remnant patches within a radius of 500–2000 m should be appropriately increased to protect plant diversity in all layers, and the negative effect of landscape heterogeneity around remnant patches, such as road disturbance, should also be considered.

## Figures and Tables

**Table 1:** Description of 16 sample sites in Guangzhou, southern China.

Vegetation Type	Sample Sites	Longitude and Latitude	Altitude (m)	Slope (°)	Aspect (°)	Dominant Species	Age of Stand (Year)	Distance from the Road (m)
Grassland	JH	113°17′09″, 23°14′31″	13	-	-	*Neyraudia reynaudiana*	≈10	50
	XG	113°16′29″, 23°12′03″	11	-	-	*Neyraudia reynaudiana*	≈10	50
	BY	113°16′08″, 23°11′34″	20	-	-	*Neyraudia reynaudiana*	≈10	50
	HSQ	113°12′38″, 23°07′52″	9	-	-	*Neyraudia reynaudiana*	≈10	50
Secondary coniferous and broad leaved mixed forest	DS	113°15′53″, 23°09′11″	59	20	280	*Pinus massoniana* -*Celtis sinensis* -*Ottochloa nodosa*	40–60	250
	YX	113°15′28″, 23°08′32″	28	10	220	*Pinus massoniana*- *Cinnamomum burmanni* -*Piper sarmentosum*	≈60	50
	TH	113°21′42″, 23°07′50″	60	8	250	*Pinus massoniana* +*Psychotria rubra* +*Ottochloa nodosa*	≈60	40
Secondary broad leaved forest	BYST	113°16′10″, 23°11′33″	180	45	13	*Schima superba* -*Psychotria rubra* -*Lophatherum gracile*	40–60	50
	BYSZ	113°18′05″, 23°10′52″	255	3	60	*Schima superba* -*Psychotria rubra* -*Lophatherum gracile*	40–60	50
	LYD	113°21′12″, 23°12′29″	60	25	230	*Schima superba* -*Psychotria rubra* -*Lophatherum gracile*	≈60	300
	PG	113°21′45″, 23°11′26″	34	5	200	*Schima superba* -*Psychotria rubra* -*Lophatherum gracile*	60–80	50
	HN	113°21′15″, 23°09′30″	60	5	210	*Schima superba* -*Cinnamomum burmanni* +*Lophatherum gracile*	60–80	250
	CL	113°28′17″, 23°12′24″	58	20	290	*Castanea henryi* *-Castanea henryi* *-Cibotium barometz*	60–100	200
	DL	113°28′58″, 23°10′21″	50	2	100	*Schima superba* -*Psychotria rubra* -*Lophatherum gracile*	>150	300
	ZS	113°29′06″, 23°08′27″	45	30	330	*Schima superba* -*Psychotria rubra* -*Adiantum capillus-veneris*	60–100	300
	WC	113°28′00″, 23°06′12″	60	30	210	*Schima superba* -*Rhaphiolepis indica* -*Dianella ensifolia*	60–100	150

**Table 2:** The selection indices of landscape heterogeneity, community structure, and topographical factors.

Types	Subtypes	Number of Indices	Indices Name
Landscape heterogeneity characteristics	Matrix indices	1	Vegetation coverage (Cv)
	Road disturbance indices	2	Road density (RD) Average distance from sample point to road (AD)
	Distance from the edge of the forest	2	Shortest distance from sample point to forest edge (SD) Farthest distance from sample point to forest edge (FD)
	Clustering indices	4	Number of patches (NP) Patch density (PD) Largest patch index (LPI) Contagion index (CONTA)
	Area-edge indices	1	Landscape shape index (LSI)
	Edge indices	2	Edge density (ED) Total edge length (TE)
	Shape indices	2	Shape Index (SHAPE) Fractal dimension index (FRAC)
	Landscape diversity indices	3	Shannon’s diversity index (SHDI) Simpson’s diversity index (SIDI) Shannon’s Evenness index (SHE)
Community structure		4	Coverage of the herb layer (Ch) Height of the herb layer (Hh) Coverage of the tree layer (Ct) Age of stand (Age)
Topographic		3	Elevation, Slope, Aspect

**Table 3:** The plant diversity indices selected in the study areas.

Types of Index	Subtypes of Index	Abbreviations	Formula	Description
Species diversity index	Shannon-Wiener index	*H*’	*H*’ *= −∑P_i_lnP_i_*	*P_i_* is the relative abundance of the *i*th species at each plot, *ln* is the natural log, and *H* describes the species richness and the equitability of individual distribution within species.
	Simpson’s index	*D*	*D = 1−∑P_i_^2^*	*P_i_* is the proportion of the individuals in species *i*, and *D* reflects the dominance in the community.
	Evenness	*J*	*J = H*’*/H*’*_max_*	*H’* is Shannon-Wiener’s biodiversity index, and *H*’*_ma_*_x_ is the maximum of *H*’.
Species richness index	Patrick Richness	R	R = S	S is the number of species in the sample plot.

**Table 4:** The dominant species in three vegetation types in Guangzhou, southern China.

Vegetation Type	Code	Dominant Herb-Layer Species (Importance Value >5%)	Dominant Shrub-Layer Species (Importance Value >5%)	Dominant Tree-Layer Species (Importance Value >5%)
Urban weeds	I	*Bidens Pilosa* (24%) *Neyraudia reynaudiana* (22%) *Themeda villosa* (6%)	-	-
Secondary coniferous and broad-leaved mixed forest	II	*Eriachne pallescens* (51%) *Pteris semipinnata* (5%)	*Psychotria rubra* (25%) *Cinnamomum burmanni* (10%) *Celtis sinensis* (6%) *Ilex asprella* var. *asprella* (6%) *Desmos chinensis* (6%) *Trema cannabina* (5%)	*Pinus massoniana* (34%) *Cinnamomum burmanni* (11%) *Celtis sinensis* (10%) *Cinnamomum camphora* (6%)
Secondary broad-leaved forest	III	*Lophatherum gracile* (22%) *Dicranopteris dichotoma* (19%) *Eriachne pallescens* (8%) *Adiantum capillus-veneris* (7%)	*Psychotria rubra* (31%) *Desmos chinensis* (5%)	*Schima superba* (45%)

**Table 5:** The multiple regression of landscape heterogeneity, community structure, and topographic factors on diversity indices and richness index in tree, shrub, and herb layers.

Layer	Biodiversity Indices	Buffer Radius/m		
		500	1000	2000
Herb	Shannon index	-Ch *, -AD	-Ch *	-Ch *
	Simpson index	AD *, -PD, Ch	Ch, RD	Ch
	Evenness	-AD *, -SLOPE *, PD *, LPI	-RD	-Ct *, Ch
	Richness	-SHAPE *, Ct *	SLOPE *, ELEVATION *, -LPI *, -ED	SLOPE, -SHAPE *, Ct *, SHDI *, AD
Shrub	Shannon index	-RD *, Ct	-NP	-LPI *, -LSI *, PD
	Simpson index	RD *, -Ct *, FD	-SLOPE *, -Ct	-SLOPE *, -Ct
	Evenness	Ct *, -FD, SLOPE	Cv *, -SHDI *, -FD	Ct *, SLOPE
	Richness	-RD *, CONTAG	AD *, -FD	-FD *
Tree	Shannon index	Ct	Ct *, -PD *, -AD *	AD,Ct
	Simpson index	-Ct	PD *, AD, -Ct	-AD, -CONTAG
	Evenness	-	-FD, -NP	-SIDI, AD
	Richness	-AGE *, NP	-AGE*	-AGE *, AD *, Cv

* indicates significant at *p* < 0.01.

## Data Availability

The data presented in this study are available on request from the author. The data are not publicly available due to privacy. Images employed for the study will be available online for readers.
